# Use of dental care services among adolescents living with HIV on antiretroviral treatment in Kampala, Uganda: a cross-sectional study

**DOI:** 10.1186/s12903-024-04426-z

**Published:** 2024-06-04

**Authors:** Maria Gorretti Nakyonyi, Nancy Birungi, Catherine Lutalo Mwesigwa, Anne Nordrehaug Åstrøm

**Affiliations:** 1 Center of International Health, University of Bergen, Bergen, Norway; 2 Institute of Odontology, University of Bergen-Department of Global Oral Health, Bergen, Norway; 3 School of Dentistry, Makerere University, Kampala, Uganda; 4 Oral Health Center of Expertise, in Vestland County, Bergen, Norway

**Keywords:** Use of dental care services, HIV, ART, Adolescents, Kampala, Uganda

## Abstract

**Objective:**

The primary purpose of this study was to assess the prevalence and socio-behavioral determinants of ever-use of dental care services among adolescents aged 10–18 years, living with HIV, on Antiretroviral treatment (ART), and attending selected HIV clinics in Kampala, Uganda.

**Methods:**

A cross-sectional study was carried out between March and September 2020. The study conveniently recruited 154 adolescents between 10 and 18 years old from 4 specific HIV clinics in Kampala. Andersen’s behavioral model guided the selection of variables, with the ever-use of dental care services as the outcome and predisposing, enabling, need-related factors and personal dental health practices as exposure variables. Data were analyzed using Fischer’s exact test for cross-tabulation and modified Poisson regression for multivariate analysis.

**Results:**

The prevalence of ever-use of dental care services was 12.3%. The adolescents aged 14–18 had higher odds of using dental care services (Prevalence ratio (PR) of 3.35 than those aged 10–13 years. Fear of the spread of HIV was negatively associated with ever-use of dental care services (PR of 0.06). Participants who were afraid of going to the dentist had higher odds of using dental care services (PR of 2.98) than those not afraid. Failure to receive dental treatment because it was not part of the medical appointment had a positive association with the ever-use of dental care services (PR of 4.50). Those who were satisfied with their dental condition had lower odds of using dental care services. The bad oral odor was positively associated with the ever-use of dental care services (PR of 2.80). The use of soap for toothbrushing was positively associated with the ever-use of dental care services (PR of 2.51).

**Conclusion:**

The study found a low frequency of dental care use among HIV-infected adolescents in Kampala, Uganda, with age being a predisposing factor. Enabling factors included fear of HIV spread, medical-dental appointment incoordination, and satisfaction with the dental condition and bad oral odor while under personal dental health practices. The use of soap for toothbrushing was an important association with dental care. Nevertheless, these study results cannot be generalized to the entire HIV adolescent population in Uganda.

**Supplementary Information:**

The online version contains supplementary material available at 10.1186/s12903-024-04426-z.

## Background

The Joint United Nations Program on HIV and AIDS (UNAIDS) reports that two of every seven new HIV (Human Immunodeficiency Virus) infections are among young people (15–24 years) [1]. The United Nations International Children’s Emergency Fund (UNICEF) reported that of the 400,010 HIV estimated incidence cases, 150,000 were adolescents aged 10–19 years [2]. . The Uganda Population-Based HIV Impact Survey (UPHIA) of 2022 indicated that the current prevalence of HIV is 1.8% among those aged between 15 and 24 years [3]. By 2020, there were approximately 6,119 incident HIV infections in the Uganda adolescent population aged 10–19, with a prevalence of about 100,000 in the same group [4]. According to the HIV Investment framework for Uganda 2021-30, approximately 65% of children between 0 and 14 living with HIV are currently on Antiretroviral treatment (ART). Among those above 15 years, about 85% were on ART as of 2019 [4].

Oral health care is an integral part of HIV care, and the spectrum of HIV-associated opportunistic diseases occurring in the oral cavity propelled dental health care providers to the forefront of patient care [5]. Over 90% of individuals living with HIV will at least have one oral manifestation attributed to HIV infection during life [6].

The most common oral manifestations include pseudomembranous candidiasis, angular cheilitis, necrotizing ulcerative gingivitis and periodontitis, oral hairy leukoplakia, Kaposi sarcoma, human papillomavirus oral warts, common ulcerative conditions, and dental caries [7–9]. Oral diseases can cause pain, discomfort, altered taste, and burning sensations, affecting daily functions like chewing, pronunciation, and socializing confidently [10].

Following the advent of ART, there has been a decrease in the prevalence of HIV-related oral lesions of 10–50% [11]. However, Scully et al. reported adverse oral health effects of antiretroviral drugs, including xerostomia, oral lichen lesions, erythema multiforme, and angular cheilitis from drugs like Didanosine and Zidovudine [12]. Xerostomia increases dental caries risk, causing oral health concerns for HIV-positive individuals [13].

Individuals living with HIV often face unmet dental care needs despite oral health issues significantly impacting their overall health and well-being [14]. Dental care has been reported to be one of the greatest unmet healthcare needs among individuals living with HIV [15]. Individuals living with HIV face a higher incidence and severity of dental disease, necessitating the need for accessible oral health care [5]. A study done in Uganda reported a caries prevalence of 80% in adolescents living with HIV taking ART compared to 67% in the general adult population [16]. Another study carried out in Uganda reported that Individuals living with HIV and taking ART had a dental treatment need of about 96% and a DMFT (Decayed, Missing, Filled Teeth) score of four [13]. People living with HIV (PLHIV) are now living longer due to ART and, hence, increased life expectancy [17]. Therefore, oral diseases are becoming increasingly important to manage in the HIV-infected population, necessitating easy access to dental care.

**Fig. 1:**
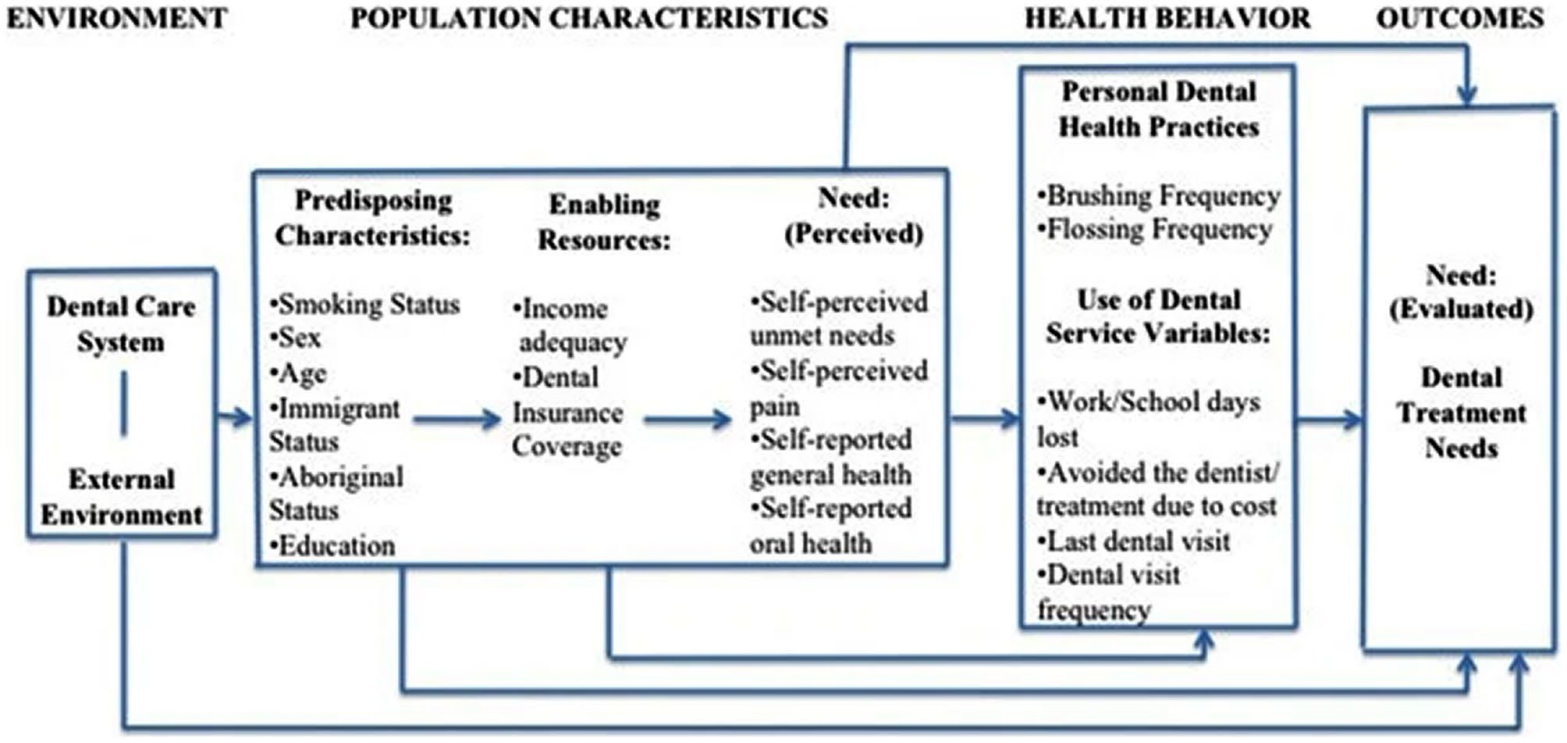
Illustration of Andersen’s modified behavioral model. Sourced from Andersen 1995 and Ramraj 2012

Andersen’s Behavioural model for health care use helps comprehend the social, personal, and systemic elements impacting the use of health services (Fig. 1). It implies that some people are more likely than others to use dental services because of need, enabling factors, predisposing factors, and personal dental health practices. These variables impact health outcomes and care satisfaction by determining the probability of individual health practices and service utilization. Andersen’s behavioral model is a standard health theoretical tool for assessing the use of health care services, including dental care services, across several studies [18–21]. Uganda’s National Minimum Health Care Package aims for Universal Health Coverage, providing preventive and curative services focusing on controlling non-communicable diseases and promoting oral health inclusivity [22]. However, despite efforts to manage specific NCDs in HIV/AIDS, the integration of oral health and HIV is still a significant concern [22]. The UHC allows free services at public facilities with user fees at private wings, but all private health facilities, including dental facilities, have 100% out-of-pocket expenditure [22]. The burden of oral health issues is exacerbated by a national policy that has not been revised in 17 years and a low budget allocation of less than 0.1% from the Ministry of Health [23]. With no HIV- oral health integration policy in the country, there is no specific budget for this. Uganda has a limited number of dentists, with only 300 serving the 44 million population. Private ownership makes dental clinics expensive, especially for marginalized groups [24]. A 2020 study revealed that 15% of Uganda’s rural areas lack a public dental facility out of 97 districts [25]. Previous studies of oral health in PLHIV conducted in Uganda have focused on the oral health-related quality of life and oral manifestations in this category of patients [26–30]. A notable proportion of Ugandans living with HIV are 10–19 years old, underscoring the significance of youth for a nation’s future [4]. In Uganda, the adolescent mark is 18 years, as anyone above that is considered an adult, hence the 10–18 age selection for this study [31]. Adolescents undergo various changes at the intellectual, developmental, and structural levels that, once modified in terms of oral health promotion, will be pivotal in an improved quality of adult life [32]. No known study has identified covariates about the ever-use of dental health care services among young people (adolescents) living with HIV in Uganda.

The primary purpose of this study is to assess the prevalence and socio-behavioral determinants of ever-use of dental care services among adolescents living with HIV on ART, between 10 and 18 years of age, attending selected HIV clinics in Kampala, Uganda.

### Methods

#### Study design

This was a cross-sectional study. It was a sub-study of a more extensive study (at this moment called parent study) entitled “*Oral health quality of life and dental treatment needs among HIV + children and adolescents on ART attending selected HIV clinics in Kampala.”*

### Study setting

The study was carried out in Kampala, the capital city of Uganda. Kampala has 1,497 health facilities, with only 57 offering ART services. Of these, only six health facilities offering ART are directly managed by the Kampala Capital City Authority (KCCA) [33]. This study selected participants from the same clinics as the parent study. These clinics had previously been purposively selected, according to the many patients receiving ART from them and the authorization of KCCA. The participants were selected from four HIV clinics: Mulago Immune Suppressive Syndrome (ISS) clinic, Kawaala Health Centre IV, Kisenyi Health Centre IV, and Kiswa Health Centre III, which acted as clusters. It was carried out between March and September 2020.

### Study participants

The inclusion criteria for this sub-study were the following: (i) Participants had to be part of the parent study; (ii) they were 10–18 years old as of March to September 2020; (iii) they were HIV positive; (iv) they had to be aware of their HIV seropositivity, (v) they had to be attending HIV clinics Kawaala, Mulago, Kisenyi, and Kiswa, (v) they were available via the telephone; and (vi) they had to be available at the time the study was conducted.

### Variables

#### Dependent variable

The dependent variable of this study was the ever-use of dental services, which was phrased as “ever-visit the dentist” in the questionnaire. It was defined as if the participant had ever visited the dentist in their lifetime.

### Independent variables

As guided by Andersen’s Behavioural model, the independent variables included *predisposing factors*: age, gender, education level, socioeconomic status, and home description. *Enabling factors*: fear of going to the dentist, knowledge of dental facility next to their residence, knowledge of dental facility next to HIV center, failure to get dental treatment because it was not part of the medical appointment, illness from other conditions, avoidance of dental care due to cost, responsible person in decision making. *Need-related factors* include overall general health, perception about teeth and mouth health, satisfaction with dental health state, need for dental treatment, dental and mouth pain, bleeding gums, and bad oral odor. *Personal dental health practices*; tooth brushing frequency and brushing materials (toothpaste, soap, salt, ash, local herbs, urine, nothing).

### Data collection, quality management, and storage

A pilot study involved 15 participants aged 10 to 18 years (not included among the 154 adolescents who officially participated in the main study). The pilot checked the questionnaire for suitability and usability. All unclear questions that confused participants were revised to aid easy comprehension. For example, questions concerning participants’ living conditions were modified to “*How would you describe the state of your home?*” Additional questions that had been developed and added to the questionnaire, like materials used for toothbrushing, i.e., local herbs, urine, ash, salt, and soap, were all piloted, aiding validation and hence adaptation for this study. On the other hand, questions like immigration status and insurance coverage were not included in the questionnaire as they did not apply to the study population.

### Interviews

This study was strictly based on telephone interviews due to the COVID-19 rules and guidelines [34]. Each interview lasted for about 30 min. A structured questionnaire that guided the interview was used using the Open Data Kit form [35] that had been standardized for all participants. Andersen’s behavioral model guided the selection of variables used to determine access and use of dental services among the selected participants [36]. Additional variables were included from previous studies and adapted to suit the Ugandan setting [21, 37, 38]. Two research assistants underwent 7- days of face-to-face training in using the Open Data Kit form, and they were also trained on how to conduct telephone interviews in both English and Luganda. The principal investigator also trained them to ask the participants different questions. These trainings were done both before and after the pilot study. They each described the study objectives and outcomes for standardization of the tool. They were given electronic tablets, Samsung Galaxy SM- T285 S# R52J60MZ22V and R52JB215 × 8TA, and trained on how to use them. Both these devices were loaded with the standardized questionnaire in an ODK form and were used uniformly for all study participants. The main data collection started following uniformity and synchrony between the two research assistants as judged by the principal investigator.

The questionnaire included socio-demographic characteristics, factors affecting the use of dental care services, personal dental health practices, general and HIV-related concerns, and attitudes, and it included the outcome variable “ever-visit—the dentist.”

Parents or caregivers were asked if the child was aware of their HIV serostatus at the start of interviews to exclude those not aware, as some parents withheld information about the serostatus and explained other reasons for frequent treatment. The questionnaire was initially constructed in English and then translated into Luganda by a local translator. Later, five independent people (the principal project study investigator, two research assistants, and two pilot study participants) reviewed the accuracy of the language translation, with language changes being made at every step. Back translation from Luganda back to English was carried out informally through analysis of questions if they meant the same in English. The questionnaire was marked reliable and valid after all five independent people agreed to the different questions without any discrepancy. The telephone interviews were conducted mainly in the locally spoken language, Luganda, and a few in English.

### Bias

An electronic data collection tool called Open Data Kit minimized human error during data collection. It had an incorporated authentication process at the point of data entry. Standardized training of research assistants was carried out to control for information bias.

### Study size

The sampling frame for this sub-study was 400 adolescents aged 10–18 who attended the four selected HIV clinics from March to September 2020. The sample size was estimated using an online calculator at a 95% confidence level and a significance level of 0.05; the calculation was based on the premise that the estimated population proportion of HIV-infected adolescents who used dental care was 50% [39]. The minimum sample size required for this study was 197 adolescents [39].

$${n'}\, = \,{{\rm{n}} \over {{\rm{1 + (}}{z^{\rm{2}}}\,{\rm{*}}\,p{\rm{(1 - }}p{\rm{))/}}{{\rm{\varepsilon }}^{\rm{2}}}N}}$$

From this formula, z is the z score, ɛ is the margin of error, N is the population size, and p is the proportion [39].

### Quantitative variables

Among predisposing factors, gender was categorized into boys (0) and girls (1). Age data was collected as a continuous variable (10–18 years). It was later dichotomized into two groups: 10–13 years (1) or 14–18 years (2). Age categories were dichotomized based on the level of intellectual capability. Most Ugandan adolescents in the 10–13 age bracket are in the primary level of education versus 14–18, who are at the secondary level, hence possible differences between the age groups. These age categories were later used to analyze enabling factors for using dental care services specific to the age groups (however, *outside of this article*). The level of education for the participants (10–18 years) had six options, and these included: Primary 1–3 (1), Primary 4–7 (2), Senior 1–4 (3), Senior 5 or 6 (4), Vocational courses (5) and those not attending school (6). These were categorized into 3: those not attending school and those with no formal school (0). Those attending primary (1–3) and primary (4–7) were categorized as attending the primary level of education (1). At the same time, those attending senior (1–4) and senior (5 or 6) were categorized under those attending secondary level of education (2). Vocational courses were categorized under the secondary level.

The socioeconomic status was assessed using a wealth index that assessed ownership of household items by Filmer et al. [40]. The participants were asked if they possessed the items or not, with categories “yes (1) or no (0)” per item. A participant was recorded as possessing the item only if the item was functioning. The items included a television, electricity, a bicycle, the availability of water, a motor car, a flush toilet, a mobile phone, a computer, a radio, a motorcycle, and a refrigerator. Five quintiles were generated using principal component analysis (PCA), with 1 representing the poorest and five the richest quintile. These were further categorized into least poor (1) and poorest (0) quintiles. The least poor category (^fourth and fifth^ quintiles) represented participants from the fairly “well-to-do-families, not necessarily rich.”

In contrast, the poorest category (1st to 3rd quintiles) represented participants from the “badly-off” families as assessed by item possession. The participants were then asked to give a perception of how they would describe their homes to assess their living conditions subjectively. The question asked was, “*How would you describe the state of your home*?” Home description had five options, which included very good (1), good (2), bad (3), very bad (4), and I do not know (5). These were categorized into two categories: good (1) for categories “*very good*” and “*good*” and bad (2) for categories “*bad*” and “*very bad*.” Those who responded, “I do not know,” could not describe the state of their home.

Under enabling factors, the questions asked were, “*Do you fear going to the dentist?*” This had three options (1–3); I do not fear (1), I fear a little (2), and very fearful (3), and these were re-categorized into 2; “yes (1)” for both little fear and very fearful and “no (0)” for no fear. Other questions asked included, *“Have you avoided dental care due to your HIV status?” “Have you avoided dental care due to fear of the spread of HIV?”* “*Have you failed to get dental treatment because it is not part of your medical appointment?” “Have you avoided dental care due to cost?”*

These questions had responses with four options (1–4), and these included yes, several times (1), yes, a few times (2), no, never (3), and I do not know (4), and these were categorized into 2; “yes (1) or no (0)”, “yes” for both several times and a few times and “no” for “no, never.” All responses with “I do not know” were added to the most frequent response.

The question, “*Who decides whether you are to see a dentist or not if you have pain in your teeth or mouth?*” had the following responses: parents (1), myself (2), my caregiver (3), my teacher (4) and I do not know (5). These were categorized into 2: parents/teachers/caregivers (0) and myself (1). Under need-related factors, questions included; “*how would you rate your health in general?*” “*How would you rate the health of your teeth and mouth?*” Both of these questions had five options: poor (1), fair (2), good (3), very good (4), and excellent (5). These were further categorized into three: poor (1), fair (2), or good (3). Good represented those that responded either “*good, very good, or excellent*.” Satisfaction with oral health state was assessed via four parameters. These included very satisfied (1), satisfied (2), dissatisfied (3), and very dissatisfied (4), and these were categorized into two options: either satisfied (1) for those that responded “*very satisfied and satisfied*” or dissatisfied (0) for those that responded “*dissatisfied and very dissatisfied*.” For the factors under personal dental health practices, the toothbrushing frequency had six responses (1–6). These included the following: never (1), several times a month (2–3 times) (2), once a week (3), several times a week (2–6 times) (4), once a day (5) and two or more times a day (6). These were then grouped into two: those who occasionally brush (0) and those who brush two or more times a day (1). Brush occasionally (0) for those who *brush once a week, several times a week, and once a day.* Those that brushed two or more times a day were categorized as (1).

Other questions included: *“What do you use to clean your teeth?”* Brushing materials assessed included soap, salt, urine, local herbs, ash, toothpaste, and nothing. These items were renamed in Excel workbook 2003(*xlsx), and each item was dichotomized into yes (1) for those who used that item for brushing or no (0) for those who did not use that item. These items were then assessed in terms of how many participants who used each brushing item reported the ever-use of dental care services. Response categories for the ever-use of dental care services were “yes” (1) and “no” (0).

Those who confirmed dental attendance were later sub-grouped and followed up with the question, “*When was your last visit to the dentist?*” Responses were given as (1) less than six months ago, (2) 6–12 months ago, (3) more than a year but less than two years ago, (4) 2–5 years ago, and (5) more than five years ago.

These were categorized into those that had visited the dentist less than a year ago (1), between 1 and 2 years ago (2), or more than two years ago (3). Reasons for the last dental visit included mandatory school check-ups or routine check-ups, emergency (tooth injury), emergency (toothache), having a tooth (teeth) pulled, filling, root canal, or others. They were renamed and dichotomized into “yes (1)” for those whose responses were positive for a particular reason or “no (0)” for those with negative responses for a reason.

The duration at the dental facility had five options, which were categorized into 3: less than 1 h (1), 1 to 2 h (2), or three or more hours (3). Average travel cost was assessed under five options (1–5). The options included the following: less than Ug. Sh. 4,000 (1), between Ug. Sh. 4100–10,000 (2), no money spent (3), more than 10,000 (4), and I do not know (5). These were categorized as follows: no money spent (0), less than Ug. Sh. 4,000 (1), between Ug. Sh. 4100–10,000 (2) or more than 10,000 (3). The oral health care provider’s treatment of the participants was also assessed, and there were five options (1–5). These included very good (1), good (2), average (3), below average (4), and do not know (5) that were categorized into 3; good (1), average (2) or poor (3). “Very good (1) and good (2)” responses were put under the “good (1)” option.

NB: All “I do not know” responses were added to the most frequent categories. (*Refer to supplementary file*)

### Statistical methods

Data were analyzed using the Statistical package Stata/SE 17.0 [41]. Categorical variables were summarized as percentages, while continuous variables were summarized with mean, medians/range. Cross-tabulations were done using Fischer’s exact test [42]. A simple modified Poisson model was used for bivariate analysis. In contrast, multivariable modified Poisson regression was used to determine factors associated with every use of dental care at a 95% confidence interval and 0.05 significance level. The measure of association was in terms of Incidence Risk Ratio (IRR) from the multivariate modified Poisson model that was interpreted as the prevalence ratio (PR) for this study. At the multivariate analysis stage, all variables related to predisposing, enabling, need-related factors, and personal dental health practices with a more likely association with the ever-use of dental care (p-value ≤ 0.2) in unadjusted analyses were included in the model.

In addition, the 4 clusters (which represented the four different clinics to which the participants belonged) were adjusted for using clustered robust standard errors incorporated in the Poisson regression model. Both checks for multicollinearity and goodness of fit of the model, including data normality, were done.

## Results

### Participants

A total of 246 adolescents were conveniently contacted according to the availability of their telephone contacts and consented to participate in a telephone interview. Of these, 44 participants either had the wrong telephone numbers or their numbers were inaccessible. These 44 participants were excluded from the study and excluded in the analysis, leaving 202 participants. However, 48 of these participants were not aware of their HIV -positive serostatus; hence, they were also excluded. The exclusion of these participants created a discrepancy in the sample size required for this study, leaving only 154 participants; hence, the participation rate was 62.6% (154/246).

### Descriptive data

Table 1 describes the total distribution of study variables according to gender. A total of 154 adolescents aged between 10 and 18 participated in this study. The data had a normal distribution, with the median and mean age being 14 and 14.3 years, respectively, while the range was 10 to 18 years. No variable had missing data in this study.

**Table 1:** Distribution of study variables in total and by gender among10-18-year-old adolescents (*n* = 154)

Variables	Total (*n* = 154)	Male (*n* = 89)	Female (*n* = 65)
**Continuous variables Median (IQR)**			
Age (years)	14(10–18)	14(10–18)	14(10–18)
**Categorical Variables frequency (%)**			
**Age categories**			
10–13	38.3	38.2	38.5
14–18	61.7	61.8	61.5
**Education level**			
No formal school	7.1	9.0	4.6
Primary level	54.6	46.1	66.2
Secondary level	38.3	44.9	29.2
**Socioeconomic status**			
Poorest	58.4	64.0	50.8
Least poor	41.6	36.0	49.2
**Home Description**			
Good	57.1	52.8	63.1
Bad	42.9	47.2	36.9
**Ever-use of dental care**			
No	87.7	87.6	87.7
Yes	12.3	12.4	12.3

### Outcome data

The prevalence of ever-use of dental care services among the study participants was 12.3%.

### Main results

Table 2 depicts the bivariate analysis of the predisposing factors of the ever-use of dental care services in terms of socio-demographic characteristics. As shown, at cross-tabulation, the ever-use of dental care services did not associate significantly with any of the predisposing factors despite the remarkable differences between the groups.

**Table 2:** Percentages of adolescents, 10–18 years, confirming the ever-use of dental care services according to predisposing factors. Fisher’s exact test (*n* = 154)

Variable	Ever visited the dentist?		*P* value
	Yes %	No %	
**Gender** Male Female	57.9 42.1	57.8 42.2	1.00
**Age** 10–13	26.3	40.0	0.32
14–18	73.7	60.0	
**Education level**			
No formal school	5.3	7.4	0.14
Primary level	36.8	57.0	
Secondary level	57.9	35.6	
**Socio-economic status**			
Poorest	57.9	58.5	1.00
Least poor	42.1	41.5	
**Home Description**			
Good	63.2	56.3	0.63
Bad	36.8	43.7	

According to Table 3, the ever-use of dental care services was statistically significantly associated with avoidance of dental care services due to the HIV status, the fear of HIV spread, failure to get dental treatment because it is not part of the medical treatment, and illness from other medical conditions.

**Table 3:** Percentages of participants confirming the ever-use of dental care services according to enabling factors, Fisher’s exact test, (*n* = 154)

Variable	Ever visited the dentist		*P*- value
	Yes %	No %	
**Afraid of going to the dentist**			
No	78.9	89.6	
Yes	21.1	10.4	0.24
**Knowledge of the Dental facility next to your residence**			
No	63.2	68.9	
Yes	36.8	31.1	0.61
**Knowledge of the Dental facility next to the HIV Centre**			
No	26.3	28.9	
Yes	73.7	71.1	1.00
**Have you ever avoided dental care due to your HIV status?**			
No	78.9	97.0	0.01
Yes	21.1	3.0	
**Have you avoided dental care due to fear of the spread of HIV?**			
No	89.5	99.3	0.04
Yes	10.5	0.7	
**Failure to get dental treatment because it is not part of the medical appointment**			
No	78.9	100.0	
Yes	21.1	0.0	0.00
**Illness from other conditions**			
No	78.9	97.8	
Yes	21.1	2.2	0.01
**Have you ever avoided dental care due to cost?**			
No	73.7	86.7	
Yes	26.3	13.3	0.17
**Responsible person in decision-making**			
Parents/caregiver/teacher	94.7	85.9	
Myself	5.3	14.1	0.47

As depicted in Table 4, the ever-use of dental care was statistically significantly associated with the following need-related factors: perception of overall health in general, perception of the health of teeth and mouth, and satisfaction with the health of mouth and teeth.

**Table 4:** Percentages of adolescents 10–18 years confirming the ever-use of dental care services according to need-related factors. Fisher’s exact test (*n* = 154)

Variable	Ever visited the dentist		*P*- value
	Yes %	No %	
**Perception about overall general health**			
Poor	15.8	3.7	
Fair	31.6	22.2	
Good	52.6	74.1	0.04*
**Perception about the health of teeth and mouth**			
Bad	36.8	6.7	
Fair	26.3	40.7	
Good	36.8	52.6	0.00**/0.002
**Satisfaction teeth**			
Dissatisfied	78.9	48.9	
Satisfied	21.1	51.1	0.02*
**Need for dental treatment.**			
No	21.1	30.4	
Yes	78.9	69.6	0.59
**Dental and mouth pain**			
No	47.4	58.5	0.46
Yes	52.6	41.5	
**Bleeding gums**			
No	68.4	77.0	0.40
Yes	31.6	23.0	
**Bad oral odor**			
No	68.4	83.0	0.21
Yes	31.6	17.0	

NB: At bivariate analysis; **p < 0.001, *p < 0.05

### Personal dental health practices

As shown in Table 5, when participants were assessed for personal dental health practices, the unadjusted analysis showed that none of the personal dental health practices was statistically significantly associated with the ever-use of dental care services.

**Table 5:** Percentages of adolescents, 10–18 years, confirming ever-use of dental care services according to personal dental health practices, Fisher’s exact test, (*n* = 154)

Variable	Ever visited the dentist		*P* -value
	Yes %	No %	
**Tooth brushing frequency**			
Brush occasionally	36.8	41.5	
Brush two or more times daily	63.2	58.5	0.81
**Brushing materials**			
**Use of soap**			
No	78.9	92.6	
Yes	21.1	7.4	0.07
**Use of salt**			
No	89.5	79.3	
Yes	10.5	20.7	0.37
**Use of local herbs**			
No	100.0	97.8	
Yes	0.0	2.2	1.00
**Use of Ash**			
No	84.2	77.0	
Yes	15.8	23.0	0.57
**Use of Toothpaste**			
No	5.3	2.2	
Yes	94.7	97.8	0.41

### Multivariate analysis

Table 6 summarizes the regression analysis of the ever-use of dental care services using modified Poisson regression models. Age, afraid of going to the dentist, failure to get dental treatment because it was not part of the medical appointment, and bad oral odour were positively associated with ever-use of dental care services. Satisfaction with teeth condition and avoidance of dental care services due to fear of the spread of HIV were negatively associated with the ever-use of dental care services. The multivariate model was tested for goodness-of-fit using a pseudo -R squared value. Pseudo -R-squared value was 26. In addition, there was no multicollinearity among variables, and the Mean Inflation Factor (VIF) was 2.54.

**Table 6:** Predisposing, enabling, need-related factors, personal dental health practices associated with the ever-use of dental care services among HIV-positive adolescents 10–18 years. Modified Poisson regression analysis (*n* = 154)

Variable	PR	95%CI	*P*-value
**Predisposing factors**			
*Gender* Male	1	1	
Female	1.43	0.74–2.78	0.28
*Age*			
10–13	1	1	
14–18	3.35	1.48–7.59	0.04
**Enabling factors**			
*Afraid of going to the dentist*			
No	1	1	
Yes	2.98	1.41–6.30	0.04
*Have you ever avoided dental care due to your HIV status?*			
No	1	1	
Yes	1.87	0.92–3.80	0.08
*Have you avoided dental care due to fear of the spread of HIV?*			
No	1	1	
Yes	0.06	0.01–0.44	0.01
*Have you ever avoided dental care due to cost?*			
No	1	1	
Yes	0.61	0.25–1.45	0.27
*Failure to get dental treatment because it is not part of the medical appointment*			
No	1	1	
Yes	4.50	1.14–17.80	0.03
*Illness from other conditions*			
No	1	1	
Yes	8.21	0.70–96.90	0.10
**Need related factors**			
*Perception about the health of teeth and mouth*			
Bad	1	1	
Fair	0.18	0.02–1.77	0.14
Good	1.08	0.60–1.95	0.79
*Perception about overall general health*			
Bad	1	1	0.59
Fair	0.82	0.39–1.72	0.06
Good	0.55	0.30–1.01	
*Satisfaction with teeth condition*			
No	1	1	
Yes	0.21	0.05–0.94	0.04
*Bad oral odor*			
No	1	1	
Yes	2.80	1.19–6.60	0.02
**Personal dental health practices**			
*Use of soap*			
No	1	1	
Yes	2.51	1.47–4.28	0.00

PR- Prevalence Ratio CI- Confidence Interval

### Frequency distribution of oral health and related characteristics of those who had ever visited the dentist

As illustrated in Table 7, tooth extraction was the main reason for the last dental visit. Participants who had ever visited the dentist were assessed on various factors related to dental care utilization, such as travel cost, time spent at the dental facility, and attitude of the dental health care providers.

**Table 7:** Percentage (n) distribution of oral health and related characteristics among those who had ever visited a dentist (*n* = 19)

Variable	Frequency *n* (%)
**Reason for the last dental visit**	
Mandatory school check-ups	
No	18 (94.7)
Yes	1 (5.3)
**Emergency toothache**	
No	7 (36.8)
Yes	12 (63.2)
**Having teeth pulled (tooth extraction)**	
No	4 (21.1)
Yes	15 (78.9)
**Filling**	
No	16 (84.2)
Yes	3 (15.8)
**Root canal**	
No	18 (94.7)
Yes	1 (5.3)
**Others**	
No	18 (94.7)
Yes	1 (5.3)
**Last dental visit**	
Less than one year	13 (68.4)
More than one year but less than two years	4 (21.1)
More than two years	2 (10.5)
**Site of dental services**	
Private	7 (36.8)
Public	12 (63.2)
**Means of transport to the dental facility**	
Walking	5 (26.3)
Bus/taxi	8 (42.1)
Bicycle	2 (10.5)
Motorcycle	4 (21.1)
**Time at the dental facility**	
Less than 1 h	2 (10.5)
Between 1 h to 2 h	4 (21.1)
More than 3 h	13 (68.4)
**Travel cost to the dental facility**	
No money spent	2 (10.5)
Less than Ug. Sh. 4,000	4 (21.1)
Between Ug. Sh. 4100–10,000	9 (47.4)
More than 10,000	4 (21.0)
**Did you feel the dental team listened well and gave you time to explain your problem**?	
No	4 (21.1)
Yes	15 (78.9)
**Has the dentist ever asked your HIV serostatus before offering dental treatment?**	
No	14 (73.7)
Yes	5 (26.3)
**Have you ever hidden/withheld your HIV status from the dentist before receiving dental treatment?**	
No	18 (94.7)
Yes	1 (5.3)
**Have you ever felt dissatisfied with the dental care you received because of your HIV status?**	
No	17 (89.5)
Yes	2 (10.5)
**Have you ever felt the dental care rendered to you was different from your friends and family whom you think do not have HIV?** No Yes	16 (84.2) 3 (15.8)
**In general, how would you describe the way your dentist treated you?** Poor Average Good	3 (15.8) 4 (21.0) 12 (63.2)

## Discussion

The study found that Ugandan adolescents living with HIV use dental care services slightly above 10%, influenced by the predisposing, enabling, need factors, and personal dental health practices as defined by Andersen’s behavioral model [36]. The study found that age, fear of dental visits, avoidance of dental care due to fear of HIV spread, failure to get dental treatment because it was not part of medical appointments, and bad oral odor, were positively associated with the ever-use of dental care services. Satisfaction with the dental condition and fear of HIV spread were negatively associated with ever-use. Andersen’s behavioral model explained 26% of the variance in dental care use, with enabling factors being more significant. Personal dental health practices were the least important in ever-use of dental care services.

The study’s strengths included exploring the use of dental care services among a vulnerable population with scarce information. It used interviews to gather data, avoiding confusion [43], and being part of a larger parent study allowed for easier recruitment and data collection. The study utilized mobile phones to address sensitive HIV and dental care questions without victimization, promoting privacy and potentially increasing response rates [44]. The Open data kit minimized human error during data collection through authentication, eliminating item non-response [45]. The study also used novel statistical methods to minimize model misspecification bias [46]. The study found that ever-use of dental care services is a less rare outcome (*prevalence greater than 10%*); hence, ordinary logistic regression could have overestimated associations. Therefore, Poisson regression was preferred over ordinary logistic regression and modified to use robust standard errors to factor in the binary outcomes [46, 47]. The study used Andersen’s behavioral model as a theoretical framework, ensuring the validity of the study variables. The 26% variance indicated a good fit for the data, but it also explains other variables as important determinants of dental care use among HIV adolescents, not included in this study.

Nevertheless, the study had some limitations. The findings could have been skewed by potential confounding factors and the nature of the study design as it did not establish causal relationships but risk indicators [48]. Andersen’s behavioral model lacks standardized variables under the different categories, making it less suitable for comparison with other studies [18]. The study’s findings may have been influenced by recall bias [49], social desirability bias, selection bias, insufficient sample size, and unit non-response, which may have compromised the accuracy of the study findings. Participants were asked about the ever-use of dental care services, which could have led to misunderstandings about childhood experiences. Previous exposure to free dental screening and emergency treatment by study participants may have overestimated ever-use prevalence. The lack of test-retest, inter and intra-examiner reliability checks for consistency over time of the questionnaire and research assistants, respectively, could have compromised the reliability of the study findings despite the vigilant training of research participants and use of an open data kit to control for inter-observer variation [50].

Additionally, the study’s selection bias could have been influenced by the inclusion of a limited number of participants, potentially compromising the precision of the findings [51]. The study’s generalizability to a larger population of adolescents with HIV and ART in Kampala or Uganda may be limited due to insufficient sample size, selection bias at both facility and individual levels, and unit non-response. Nevertheless, the selection of HIV centers was based on ART coverage [33].

There have been variations reported in the prevalence of use of dental care services among the HIV population, contrary to the 12.3% reported in this study. With 19%, 8%, 9%, and 18.5% in China, Nigeria, Uganda, and Tanzania respectively [13, 52–54]. The differences could be attributed to differences in study populations, i.e., adolescent versus adult HIV populations, economic situations, dental accessibility, and health financing systems in the different regions [55, 56]. Similar to this study, some studies have reported an association between older age and better oral health care retention [57]. Inconsistent with this study, previous studies have shown a negative association between fear of dentists and utilization of dental care services among adults living with HIV [53, 58–60]. These differences may be attributed to age and cultural variations. Similar to this study, a Sudan study reported that 75% of dental patients fear HIV transmission at dental facilities [21]. A study in Florida also found that adults with HIV experienced treatment fatigue due to overwhelming appointments with doctors and dentists [59]. Another study in Northern California found that 21% of women living with HIV failed to book dental appointments, and poor oral health perception had a negative influence on the use of dental care services [60]. Studies have shown that dental condition satisfaction positively influences dental care service use in the general adult population of unknown HIV status, not necessarily the HIV adolescent population [37]. Other similar findings from other studies include extractions for dental caries management, with emergency toothache being the primary reason for visiting the dentist, long waiting times for appointments, and discrimination concerns due to HIV [21, 59, 61, 62].

This study’s low utilization rate of dental services may be due to limited awareness and availability of dentists [24]. Many patients may be unaware of the dental services due to a lack of an HIV dental integrated program in Uganda. Additionally, dental clinics are often privately owned, making them expensive for adolescents living with HIV [25]. The perception of premature death or a short life span also contributes to low levels of dental care use [59]. Concerning age, the older the individual, the longer they are exposed to the adverse effects of ART, potentially leading to high dental caries rates [16]. Most participants only visit the dentist as an emergency; therefore, fear of dental visits may also contribute to poorer oral health and a greater need for dental services [63, 64]. Lack of trust in and knowledge about infection control systems, such as not washing hands, using protective eyewear, and sterilizing instruments, can lead to fear of HIV spread in dental facilities [65]. The lack of coordination between medical and dental appointments could reveal that dental care services are primarily used for emergencies. This, combined with frequent medical care for HIV patients, can make it difficult to balance oral health needs, negatively impacting school attendance and income for caregivers and parents. The lack of pain and toothache could deter the use of dental care services as it brings false satisfaction with the oral health status. Conversely, bad oral odor negatively affects HIV patients’ quality of life, possibly due to ART-induced xerostomia and oral lesions [12, 66]. The study findings may suggest that soap lacks fluoride and hence cannot prevent dental caries. This leads to increased dental service utilization in patients due to the frequency of dental caries in soap participants. The study reveals that many individuals in Uganda are unaware of treatment options and have limited access to preventive treatments, leading to a lack of regular dental care services. This is due to financial status and perception of the relevance of dental treatment. Dental and mouth pain often present as emergencies, leading to a focus on treatment rather than prevention [19]. This low priority for oral health negatively affects quality of life, leading to delayed dental visits.

The study also found that the cost of dental care services significantly influences the type of facility participants visit. Most dental patients sought care from public facilities and used public transport, a cheap alternative for many Ugandans. This could be a barrier to seeking dental services in urban settings like Kampala, as low socioeconomic status individuals are more likely to be infected with HIV [67]. Long waits at the dental visit may be due to the large patient-provider ratio. The study reveals that despite HIV education, stigma and discrimination persist among HIV-positive individuals, leading to a negative attitude from dental care providers. This fear of rejection and breach of confidentiality may deter them from seeking dental services. The findings provide a basis for further research on HIV-related dental experiences in Uganda.

The study suggests the necessity of public health campaigns to enhance patient dental awareness, promote the use of oral health programs, and alleviate dental care anxiety [58, 68]. It also emphasizes the importance of good oral health for HIV-positive individuals, emphasizing the need for synchronization between medical and dental appointments, research on HIV stigma, and investment in dental practitioner training [69].

## Conclusion

The study found a low frequency of dental care use among HIV-infected adolescents in Kampala, Uganda, with age being a predisposing factor. Enabling factors included fear of HIV spread, medical-dental appointment incoordination, and satisfaction with the dental condition and bad oral odor while under personal dental health practices. Using soap for toothbrushing was an important association with dental care use. Nevertheless, these study results cannot be generalized to the entire HIV adolescent population in Uganda.

### Electronic supplementary material

Below is the link to the electronic supplementary material.

Supplementary Material 1

## Data Availability

The datasets used and/or analyzed during the current study are available from the corresponding author on reasonable request.

## Abbreviations

AIDS: Acquired Immunodeficiency Syndrome
ART: Antiretroviral treatment
DMFT/dmft: Decayed, missing, filled teeth
HIV: Human Immunodeficiency Virus
KCCA: Kampala Capital City Authority
MJAP: Makerere University Joint AIDS Program
NCDs: Non-Communicable Diseases
PCA: Principal Component Analysis
PLHIV: People Living with HIV
UPHIA: The Uganda Population-Based HIV Impact Survey
UNAIDS: Joint United Nations Program on HIV and AIDS
IRR: Incidence Rate Ratio
PR: Prevalence Ratio
CI: Confidence Interval
MakSHSREC: Makerere University Institutional Review Board, School of Health Sciences Research and Ethics Committee
UHC: Universal Health Coverage
UNCST: Uganda National Council of Science and Technology
REK: Norwegian Regional Ethics Committee
