# Supplementary material for: Use of dental care services among adolescents living with HIV on antiretroviral treatment in Kampala, Uganda: a cross-sectional study
Source: BMC Oral Health. 2024 Jun 4;24:654. doi: 10.1186/s12903-024-04426-z (PMC11149271; doi:10.1186/s12903-024-04426-z)
Supplement: Supplementary file 1 — Supplementary Material 1 [file 12903_2024_4426_MOESM1_ESM.docx]

**Table A1 showing the questionnaire variables and scores before and after categorization**

| **Question variable** | **Initial score** | **Categorized score** |
| --- | --- | --- |
| **Predisposing factors** |  |  |
| Gender | 0-boy  1-girl | (0)-boy  (1)-girl |
| Age |  | (1)-10-13 years  (2)-14-18 years |
| Which class are you? | 1-Primary 1-3  2-Primary 4-7  3-Senior 1-4  4-Senior 5 or 6  5-Vocational courses  6-not attending school | (0)-no formal school (6)  (1)-primary level of education (1,2)  (2)-secondary level of education (3,4,5) |
| Do you have any of the following things in your home and do they work? | 1-Television  2-Electricity  3-Bicycle  4-water  5-Motor car  6-Flush toilet  7-Mobile phone  8-Computer  9-Radio  10-motorcycle  11-Refrigerator | 1-Television (yes (1), no (0))  2-Electricity (yes (1), no (0))  3-Bicycle (yes (1), no (0))  4-Water (yes (1), no (0))  5-Motor car (yes (1), no (0))  6-Flush toilet (yes (1), no (0))  7-Mobile phone (yes (1), no (0))  8-Computer (yes (1), no (0))  9-Radio (yes (1), no (0))  10-Motorcycle (yes (1), no (0))  11-Refrigerator (yes (1), no (0)) |
| How would you describe the state of your home? | 1-very good  2-good  3-bad  4-very bad  5-I don't know | (1)-good (1,2)  (2)-bad (3,4) |
| **Enabling factors** |  |  |
| Do you fear going to the dentist? | 1-I do not fear  2-I fear a little  3-very fearful | (1)-yes (2,3)  (0)-no (1) |
| Have you avoided dental care due to your HIV status? | 1-yes, several times  2-yes, a few times  3-no, never  4-I do not know | (1)-yes (1,2)  (0)-no (3) |
| Have you avoided dental care due to fear of spread of HIV? | 1-yes, several times  2-yes, a few times  3-no, never  4-I do not know | (1)-yes (1,2)  (0)-no (3) |
| Have you failed to get dental treatment because it isn't part of your medical appointment? | 1-yes, several times  2-yes, a few times  3-no, never  4-I do not know | (1)-yes (1,2)  (0)-no (3) |
| Have you avoided dental care because of illness from other medical conditions?  Have you avoided dental care due to cost? | 1-yes, several times  2-yes, a few times  3-no, never  4-I do not know | (1)-yes (1,2)  (0)-no (3) |
| Do you know of any dental facility near your home? | 1-yes  0-no | (1)- yes  (0)-no |
| Do you know of any dental facility near where you receive your HIV care? | 1-yes  0-no | (1)- yes  (0)-no |
| Who decides whether you are to see a dentist or not if you have pain in your teeth or mouth? | 1-parents  2-myself  3-my caregiver  4-my teacher  5-I do not know | (1)-myself (2)  (0)-parents/teachers/caregivers (1,3,4) |
| **Need-related factors** |  |  |
| How would you rate your health in general? | 1-poor  2-fair  3-good  4-very good  5-excellent | (1)-poor (1)  (2)-fair (2)  (3)-good (3,4,5) |
| How would you rate your health of teeth and mouth? | 1-poor  2-fair  3-good  4-very good  5-excellent | (1)-poor (1)  (2)-fair (2)  (3)-good (3,4,5) |
| Are you satisfied/ happy with the health of your teeth or mouth? | 1-very satisfied  2-satisfied  3-dissatisfied  4-very dissatisfied | (1)-satisfied (1,2)  (0)-dissatisfied (3,4) |
| Do you think/feel you need dental treatment? | 1-yes  0-no  2-I don’t know | (1)- yes  (0)-no |
| Do you have pain because of your teeth or mouth? | 1-yes  0-no | (1)- yes  (0)-no |
| Do you have bleeding gums? | 1-yes  0-no | (1)- yes  (0)-no |
| Do you have bad smell coming from your mouth? | 1-yes  0-no | (1)- yes  (0)-no |
| **Personal dental health practices** |  |  |
| How often do you brush your teeth? | 1-never  2-several times a month (2-3 times)  3-once a week  4-several times a week (2-6 times)  5-once a day  6-2 or more times a day | (1)-Brush 2 or more times daily  (0)- Brush occasionally |
| What do you use for cleaning your teeth? | 1-soap  2-salt  3-urine  4-local herbs  5-ash  6-toothpaste  7-nothing | 1-soap (yes (1), no (0))  2-salt (yes (1), no (0))  3-urine (yes (1), no (0))  4-local herbs (yes (1), no (0))  5-ash (yes (1), no (0))  6-toothpaste (yes (1), no (0))  7-nothing (yes (1), no (0)) |
| **Those that had ever visited the dentist** |  |  |
| When was your last visit to the dentist? | 1-less than 6 months ago  2-6-12 months ago  3-more than a year but less than 2 years ago  4-2-5 years ago  5-more than 5 years ago | (1)-less than a year ago (1,2)  (2)-between 1-2 years ago (3)  (3)-more than 2 years ago (4,5) |
| Where did you receive dental services? | 1-private clinic  2-public health centre |  |
| What was the reason for your last dental visit? | 1-mandatory school check-ups / routine check-ups  2-emergency (tooth injury)  3-emergency (toothache)  4-having tooth (teeth) pulled 5-filling  6-root canal  7-others | 1-mandatory school check-ups / routine check-ups (yes (0), no (0))  2-emergency (tooth injury) (yes (0), no (0))  3-emergency (toothache) (yes (0), no (0))  4-having tooth (teeth) pulled (yes (0), no (0))  5-filling (yes (0), no (0))  6-root canal (yes (0), no (0))  7-others (yes (0), no (0)) |
|  |  |  |
| Which means of transport did you use to access the preferred dental facility? | 1-private car  2-walking  3-bus/taxi  4-bicycle  5-motorcycle | 1-private car (yes (0), no (0))  2-walking (yes (0), no (0))  3-bus/taxi (yes (0), no (0))  4-bicycle (yes (0), no (0))  5-motorcycle (yes (0), no (0)) |
| How much time did you spend at the dental facility from arrival to the time you left after treatment? | 1-Less than 1 hour  2-1 to 2 hours  3-3 to 4 hours  4-Greater than 4 hours  5-I do not know | (1)-less than 1 hour (1)  (2)-1 to 2 hours (2)  (3)-3 and more hours (3,4) |
| What was the average travel cost to and from the dental clinic? | 1-less than Ug. Sh. 4,000  2-between Ug. Sh. 4100 – 10000  3-no money spent  4-more than 10000  5-I do not know | (0)-no money spent  (1)-less than Ug. Sh. 4,000  (2)-between Ug. Sh. 4100 – 10000  (3)-more than 10000 |
| Do you feel the dental team listened well and gave you time to explain your problem? | 1-yes  0-no | (1)- yes  (0)-no |
| Has the dentist ever asked for your HIV status before offering dental treatment? | 1-yes  0-no | (1)- yes  (0)-no |
| Have you ever hidden/withheld your HIV status from the dentist before receiving dental treatment? | 1-yes  0-no | (1)- yes  (0)-no |
| Have you ever felt not satisfied with the dental care you received because of your HIV status? | 1-yes  0-no | (1)- yes  (0)-no |
| Have you ever felt the dental care rendered to you was different from your friends and family whom you think do not have HIV? | 1-yes  0-no | (1)- yes  (0)-no |
| In general, how would you describe the way your dentist treated you? | 1-very good  2-good  3-average  4-below average  5- I don't know | (1)-good  (2)-average  (3)-poor |

**NB: All “I don’t know” responses were placed under the most frequent categories.**
